# Hypercalcemia as a biomarker of myeloproliferative neoplasms?

**DOI:** 10.17179/excli2019-1765

**Published:** 2019-09-03

**Authors:** Stephen E. Langabeer

**Affiliations:** 1 Cancer Molecular Diagnostics, St. James's Hospital, Dublin, Ireland

## ⁯⁯⁯

***Dear Editor*,**

Hypercalcemia of malignancy (HCM) is a paraneoplastic syndrome estimated to occur in up to 30 % of cancer patients. The two major types of HCM differ in their pathophysiology: humoral, whereby tumors secrete parathyroid hormone-related protein that promotes increased osteoclast activity and bone resorption with subsequent release of calcium, and secondly, osteolytic HCM due to excessive calcium release from bone secondary to malignant invasion of the bone marrow. Humoral HCM is associated with renal, ovarian, breast and squamous cell carcinomas whereas osteolytic HCM is often observed in malignancies with bone metastasis, in addition to breast cancer and myeloma (Feldenzer and Sarno, 2018[2]).

The diagnostic hallmark of the myeloproliferative neoplasm (MPN) of chronic myeloid leukemia (CML) is the Philadelphia (Ph) chromosome resulting in the *BCR-ABL1* fusion gene, whereas the most commonly acquired genetic abnormality in the Ph-negative MPN of polycythemia vera, essential thrombocythemia and primary myelofibrosis is the *JAK2* V617F mutation. In addition to these molecular features, additional clinico-pathological criteria are necessary for the diagnosis and classification of these MPN (Arber et al., 2016[1]). Despite these criteria, hypercalcemia in the absence of other clinical or laboratory features of an MPN has become a periodic, if infrequent prompt for molecular testing of the *BCR-ABL1* fusion and/or the *JAK2* V617F.

In order to address the clinical and laboratory value of such requests, a retrospective audit was performed on all diagnostic *BCR-ABL1* and *JAK2* V617F requests received at a molecular diagnostics center for hematological malignancies from January 2006 to June 2019 inclusive. Of 20,086 and 9,570 diagnostic requests for the *JAK2* V617F mutation and *BCR-ABL1* fusion respectively, 59 requests (0.2 %) were received for investigation of either* JAK2* V617F (n=35), *BCR-ABL1 *(n=12) or both (n=12), with the sole clinical details provided of hypercalcemia. The median age was 63 years and comprised 34 males and 25 females. Using standardised assays, the *JAK2* V617F mutation or *BCR-ABL1* fusion were not detected in any of the 59 patients.

Which patients to screen for MPN-associated rearrangements and mutations requires scrutiny in order to optimise laboratory resources, however the number of requests with hypercalcemia did not adversely affect laboratory workload. Acknowledging that HCM has been previously reported in rare instances of CML and MPN (Khoury et al., 2012[3]; Toro-Tobón et al., 2017[4]), the rationale for reflexive screening of the *BCR-ABL1* and *JAK2* V617F rearrangements in patients presenting with hypercalcaemia without other evidence of an MPN is unfounded. 

## Conflict of interest

The author declares no conflict of interest.
